# Urban Wastewater Metagenomics Reveals the Antibiotic Resistance Gene Distribution Across Latvian Municipalities

**DOI:** 10.3390/microorganisms14010145

**Published:** 2026-01-09

**Authors:** Edgars Liepa, Maija Ustinova, Dita Gudra, Ance Roga, Ineta Kalnina, Brigita Dejus, Sandis Dejus, Martins Strods, Laura Elīna Tomsone, Juris Kibilds, Vadims Bartkevics, Aivars Berzins, Uga Dumpis, Talis Juhna, Davids Fridmanis

**Affiliations:** 1 Latvian Biomedical Research and Study Centre, LV-1067 Riga, Latvia; edgars.liepa@biomed.lu.lv (E.L.); maija.ustinova@biomed.lu.lv (M.U.); dita.gudra@biomed.lu.lv (D.G.); ancer@biomed.lu.lv (A.R.); ineta@biomed.lu.lv (I.K.); 2 Water Systems and Biotechnology Institute, Riga Technical University, Kipsalas Street 6a, Kurzemes Rajons, LV-1048 Riga, Latvia; brigita.dejus@rtu.lv (B.D.); sandis.dejus@rtu.lv (S.D.); martins.strods_4@rtu.lv (M.S.); talis.juhna@rtu.lv (T.J.); 3 Institute of Food Safety, Animal Health and Environment, BIOR Lejupes Street 3, LV-1076 Riga, Latvia; laura.tomsone@bior.lv (L.E.T.); juris.kibilds@bior.lv (J.K.); vadims.bartkevics@bior.lv (V.B.); aivars.berzins@bior.lv (A.B.); 4 Pauls Stradins Clinical University Hospital, Pilsonu Iela 13, Zemgales Priekspilseta, LV-1002 Riga, Latvia; uga.dumpis@gmail.com

**Keywords:** metagenomics, wastewater-based epidemiology (WBE), antibiotic resistance, resistome, Latvia

## Abstract

Antimicrobial resistance (AMR) poses a global health threat, with urban wastewater systems serving as key reservoirs for resistance dissemination. This study aimed to investigate the relationships among urban environments, bacterial communities, and AMR patterns, and evaluate the specific municipal-scale drivers of resistance gene distribution. Shotgun metagenomic analysis was conducted on 45 wastewater samples collected from 15 municipalities across Latvia to determine the composition of the resistome and its correlation with local factors. The analysis identified 417 distinct antibiotic resistance genes (ARGs) belonging to 108 families, with geographic location serving as the primary driver of ARG distribution, which explained 65.87% of community variation (*p* = 0.001). Local industrial factors demonstrated significant effects, with food industry wastewater significantly influencing both bacterial taxonomy and ARG profiles (*p* < 0.05). While the presence of a regional hospital did not shape the overall municipal resistome, hospital-associated wastewater showed 19 overlapping ARGs, including clinically critical carbapenemases. Municipal wastewater systems function as geographically structured reservoirs of AMR that are shaped by localized industrial and healthcare outputs. These findings support wastewater-based AMR surveillance as a valuable tool for tracking specific resistance sources.

## 1. Introduction

Antimicrobial resistance (AMR) occurs when infection-causing microorganisms, known as pathogens, evolve to survive antimicrobial treatments, such as antibiotics [1]. However, human activities have accelerated this process through antimicrobial overuse and misuse across the healthcare, agricultural, and veterinary sectors. Due to its rapid emergence, AMR has been identified by the World Health Organization (WHO) as one of the most prominent threats to global health. Without effective antimicrobials, our ability to treat infectious diseases is severely compromised, leading to prolonged illness, increased risk of disease spread, and elevated mortality rates. Furthermore, AMR threatens the health of plants and animals, potentially causing significant downstream effects on agricultural productivity, food security, and the economy [1,2].

The threat of antibiotic resistance requires increasing global efforts to monitor and limit the spread of antibiotic-resistant bacteria. Wastewater (WW) often contains hazardous contaminants, including pharmaceuticals, pathogens, and bacteria carrying antibiotic resistance genes (ARGs) [3,4,5,6]. This environment facilitates the transfer of antibiotic-resistant bacteria and genes among humans, animals, and the surrounding environment [7,8,9,10]. Previous studies have demonstrated that WW composition can vary between geographical locations [11], depending on the origin of the WW, particularly whether it originates from households, industrial factories or rainwater drained from streets, house roofs, or other surfaces. Specific production industries, such as pharmaceutical manufacturing, metallurgy, and animal farming, have considerable impacts on WW composition by discharging large amounts of waste or toxic byproducts [12,13,14,15].

Although WW management practices have vastly improved, microorganisms from sewage systems are still being found in natural water bodies, especially near urban areas [16,17,18,19]. Microorganisms may enter natural water systems through exceptional events such as sewage overflows due to extreme precipitation, and damage to sewer pipes. Treated WW effluent is another source. Although it contains fewer living organisms than untreated WW, it can still alter natural water environments [20,21], caused by the biological and chemical agents remaining in the effluent after treatment [3,4,5,6,16,19]. Therefore, investigating WW composition as a potential pollution source has become increasingly relevant.

Next-generation sequencing (NGS) and metagenomics have emerged as powerful tools for studying ARGs [22,23]. Technological advancements and the COVID-19 pandemic have accelerated the wider adoption of WW-based epidemiology (WBE), offering insights into societal habits and disease trends [22,23,24,25]. While global initiatives have begun to map the urban resistome, significant geographic disparities in data availability remain. Central and Western Europe have established relatively robust environmental monitoring networks [11,16,24,25], but Latvia has been underrepresented in these global datasets.

Latvia presents a unique ecological and demographic context for AMR surveillance. Unlike the densely populated and heavily industrialized regions of Central Europe, Latvia is characterized by a lower population density, a distinct industrial profile dominated by food processing and timber, and specific patterns of antibiotic consumption. While previous studies in the Baltic Sea region have detected macrolide and fluoroquinolone resistance genes in coastal waters and sediments [26], the upstream sources of this contamination remain poorly characterized. Therefore, a systematic, high-resolution analysis of antibiotic resistance determinants in municipal wastewater in Latvia is critical. Filling this knowledge gap is essential not only for local public health but also for understanding the role of Baltic municipalities as reservoirs for resistance.

In this study, we characterized the microbial community and resistome signatures in WW from 15 Latvian municipalities using short-read metagenomic sequencing of untreated samples. We conducted taxonomic profiling and performed metagenomic assembly to assess the distribution and diversity of ARGs. City WW systems, with their continuous inflow of organic matter, constant temperature, and high microbial density, constitute biologically active environments that support robust, relatively stable microbial communities. We expect that the stability and mixing inherent to municipal WW environments will facilitate the persistence and dissemination of ARGs, with distinctive resistome profiles emerging in cities with substantial hospital or industrial contributions.

## 2. Materials and Methods

### 2.1. Sample Collection

**Figure 1:**
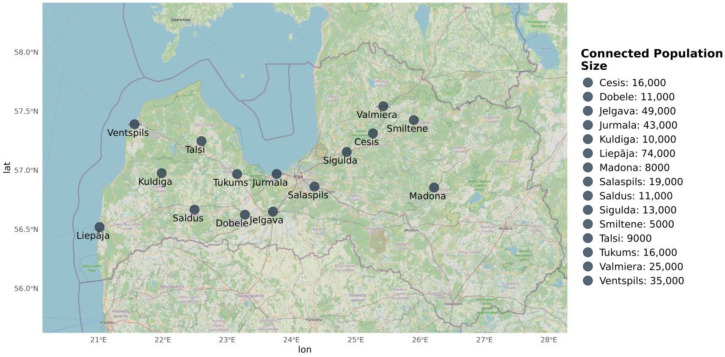
Sampling locations and size of connected population of the WW system.

Untreated WW samples were collected from 15 municipalities in Latvia (Figure 1) between August and November 2020. Sampling was conducted once a week for three weeks at each site, resulting in a total of 45 samples (one sample per site per week). A portable autosampler Sigma SD900 (Hacha, Loveland, CO, USA) operating in time-dependent mode (300 mL/h) was used to collect 24 h composite raw WW samples (7.2 L) at the inlet of the WW treatment plant (WWTP). Samples were immediately transferred to the laboratory, stored at 4 °C, and further processed within 24 h.

Municipalities were classified based on population size connected to WW systems (Table 1) and the presence and type of healthcare institutions (level 0 to 4 hospitals, including specialized facilities, Red Cross hospitals, and branch institutions) according to the Latvian National Health Service classification [27].

The industrial WW impact was classified as the percentage of industrial WW in the total WW flow reported from each city. The categories were defined as:

• High: ≥30%
• Medium: 15–29.9%
• Low: 1–14.9%
• None: 0%
• Seasonal: Applied to cities with significant seasonal variations.

To evaluate the impact of the food industry while accounting for city size, we created a weighted Food Industry Impact Index (FIII). First, the proportional volume of food industry wastewater was calculated:

(1) $$Food\;Industry\;WW\;Proportion\;\left(\%\right)=\;\frac{Food\;Industry\;WW\;Volume}{Total\;WW\;Volume}\;\times \;100$$

To balance the relative impact between large and small cities, a weighting factor (*WF*) was calculated:

(2) $$WF=\frac{1}{\;\sqrt{Total\;WW\;Volume}}$$

The *WF* decreases as the total *WW volume* increases, ensuring that smaller cities receive proportionally more weight. The final *FIII* was calculated as follows:

(3) FIII=Food Industry WW Proportion (%) × WF

Allowing for comparison across cities of varying sizes. This combines the food industry’s contribution with the WF to balance the influence of city size. The resulting categories were:

• High Impact: *FIII* > 0.3
• Medium Impact: 0.1 ≤ *FIII* ≤ 0.3
• Low Impact: 0 < *FIII* < 0.1
• None: *FIII* = 0
• Seasonal: Cities with seasonal variations (e.g., Jūrmala).
• NA: For cities with missing or incomplete data.

Additional binary classifications (presence/absence) were established for specific industrial sectors present in the municipality: food processing, dairy production, meat production, metal processing, and automotive washing facilities connected to municipal WW systems.

### 2.2. DNA Extraction and Metagenomic Sequencing

Microbial DNA was isolated from concentrated WW using the FastDNA SPIN Kit for Soil (MP Biomedicals, Eschwege, Germany) according to the manufacturer’s guidelines. DNA was normalized to 500 ng and sheared using a Covaris S220 Focused-ultrasonicator (Covaris, Woburn, MA, USA) to achieve an average fragment size of 400 bp. Libraries were constructed using MGIEasy Universal DNA Library Prep Set V1.0 (MGI Tech Co., Shenzhen, China). Quality control was performed using the Qubit High Sensitivity dsDNA assay kit on a Qubit 2.0 instrument and using the Agilent High Sensitivity DNA kit on an Agilent 2100 Bioanalyzer (Agilent Technologies, Santa Clara, CA, USA). Sequencing was performed on a DNBSEQ-G400 sequencer using DNBSEQ-G400RS High-Throughput Sequencing Set (MGI Tech Co., Shenzhen, China) according to the manufacturer’s guidelines to a depth of at least 20 million paired-end reads (150 bp) per sample.

### 2.3. Metagenomic Data Analysis

Quality trimming of the obtained raw paired-end reads was performed by Trimmomatic v.0.39 [28] (parameters: LEADING:Q30, TRAILING:Q30, MINLEN:36). Human host sequences were removed by mapping against the hg19 reference genome using Bowtie2 v.2.4.2 [29]. Unmapped read pairs were extracted using SAMtools v1.9 [30] and then divided into paired-end read files by bedtools2 v.2.18 [31]. Taxonomic profiling was performed using Kraken2 [32] with the RefSeq release 98, and taxonomic agglomeration was performed using the kraken-biom package [33].

De novo read assembly was performed using IDBA-UD [34] assembler with a k-mer length of at least 50. Assembly quality was evaluated using metaQuast [35]. To determine coverage, host-removed reads were mapped back to the assemblies using Bowtie2 (--sensitive-local) and processed with SAMtools v1.9.

### 2.4. Metagenome-Assembled Genome and Mobile Genetic Element Reconstruction

Metagenome assembled genome (MAG) and mobile genetic element (MGE) reconstruction was performed using EBI assembly, binning and annotation pipelines. De novo assembled contigs were generated with the miassembler pipeline v1.0.0 [36], which orchestrates read quality control (fastp v0.23.4 [37] phred quality ≥ Q15, unqualified percent limit 40%), decontamination of human and PhiX sequences using BWA-MEM2 v2.2.1 [38], and metagenomic assembly with MEGAHITv1.2.9 [39] assemblers (min K-mer size 21, max K-mer size 99, k-step 20, contig length ≥ 200). Assembly quality and coverage statistics were generated using QUAST v5.2.0 [40], SeqKit v2.9.0 [41], MultiQC v1.25.1 [42], and jgi_summarize_bam_contig_depths scripts [43]. QC-filtered and assembled contigs were then used as primary input for binning, genome annotation, and mobile genetic element reconstruction.

MAG reconstruction was performed using the genomes-generation pipeline v1.1.0 [44]. The assembly-derived contigs were then binned using three complementary algorithms: MetaBAT2 v2.15 [45], MaxBin2 v2.2.7 [46], and CONCOCT v1.1.0. Resulting bins were refined using the mgbinrefinder subworkflow. Bin quality and taxonomic assessment included CAT [47], GUNC v4 [48], CheckM2 v1.0.1 [49], and dereplication with dRep v3.2.2 [50]. Bins were further evaluated for rRNA and tRNA content using cmsearch v1.1.4 [51] and assigned taxonomy using GTDB-Tk v2.2.6 [52,53]. High-quality MAGs were defined as those meeting thresholds of >90% completeness and <5% contamination.

Mobile genetic element identification was performed with the EBI mobilome-annotation pipeline v2.0.2 [54]. This pipeline integrates several specialized tools for comprehensive MGE annotation, including AMRFinderPlus v3.11.4 [55] (database version 2023-02-23.1), Diamond v2.0.12 [56], geNomad v1.11.1 [57], ICEfinder v1.0 [58], IntegronFinder2 v2.0.6 [59], ISEScan v1.7.3 [60], MobileOG-DB Beatrix 1.6 v1 [61], PROKKA v1.14.6 [62], and VIRify v3.0.0 [63]. Plasmids were validated using PlasFlow v1.1.0 [64] and PlasmidFinder v2.1.6 [65,66].

### 2.5. Detection of ARGs

ARGs from de novo metagenome contigs generated by IDBA-UD were identified using RGI version 6.0.3 against the Comprehensive Antibiotic Resistance Database (CARD) release 2 October 2023 [67], restricting analysis to high-confidence hits (Best_Identity > 90%). For MGEs and MAGs, nf-funcscan version 2.0.0 [68] annotated ARGs and associated functional genes from assembled contigs, with a 90% sequence identity threshold. Quality-controlled reads were mapped using BWA-MEM2 v2.2.1, and ARG coverage was determined by HTSeq-count v.2.0.3 [69] in union mode.

### 2.6. Normalization and Statistical Analysis

Statistical analysis was conducted in R version 4.4.0 [70] using the phyloseq package version 1.48.0 [71] to process ARG and taxonomic abundance data. Initially, the distribution of reconstructed taxa and ARG counts per sample was assessed to identify and remove outliers. The sequencing depth differences between samples were evaluated, confirming that the maximum variation did not exceed ten-fold. To assess how sequencing depth might influence the detection and recall of ARGs, rarefaction curves were generated using the *rarecurve* () function from the vegan package version 2.7-1 [72].

Alpha diversity metrics (Shannon, Inverse Simpson) were calculated with *estimate_richness* () function from the phyloseq package using unnormalized and unfiltered ARG counts. This approach preserves rare features such as singletons and doubletons as recommended by phyloseq tutorials [73]. Differences between groups were statistically evaluated using the Kruskal–Wallis rank sum test for multi-level factors and the Wilcoxon rank-sum test for binary comparisons from the R package stats v.4.4.2. Post hoc pairwise comparisons for significant Kruskal–Wallis results were performed with Dunn’s test applying Benjamini–Hochberg correction to control false discovery rate.

For beta diversity, singleton ARGs were removed to reduce noise, and counts were normalized using Cumulative Sum Scaling (CSS) using the normalize function in the microbiomeMarker package version 1.10.0 [74] to correct for differences in sequencing depth and compositional effects across samples. Non-rarefied, CSS-normalized count data were used for ordination and statistical testing to preserve the integrity of the relative abundance information. Ordination was visualized using Non-metric Multidimensional Scaling (NMDS) based on Bray–Curtis dissimilarity. Differences in community structure were tested using PERMANOVA with the *adonis*2 function from the vegan package. Multivariate homogeneity of group dispersions, a prerequisite for valid PERMANOVA interpretation, was confirmed using the *betadisper* function from the same package.

For the identification of differentially abundant ARGs associated with environmental or categorical variables, the SIAMCAT v2.12.0 [75] framework was utilized. Analysis was performed on the total sum scaled normalized data derived from the phyloseq object. This method enables robust supervised machine learning and statistical association testing tailored for microbiome compositional datasets.

### 2.7. Core Elements

Core microbiome membership was determined using the Microbiome Analytics R package [76]. Taxa or genes were considered part of the core microbiome if they were consistently detected in at least 70% of samples (prevalence threshold), with a minimum relative abundance of 0.1% for taxonomic features. For ARGs, core status required detection of more than one gene copy per sample. This framework integrates both abundance and occupancy (prevalence) data, following established methods for ecological synthesis of microbiome data [77].

## 3. Results

The wastewater samples yielded high-quality sequencing data that facilitated a robust de novo metagenome assembly suitable for ARG identification. Sequencing produced an average depth of 23 million 150 bp paired-end reads per sample, with a final retention rate of 99.97% after quality trimming and host sequence removal (Table S1 *sequencing_reads_par_sample.tsv*). Input sequences generated over 3 million contigs longer than 1000 bp, characterized by an N50 of 1426 bp and an average length of 2397 bp. The assembly demonstrated significant continuity, producing 62,572 scaffolds longer than 10,000 bp and a maximum contig length of 956,861 bp, with an average duplication ratio of 1.24 (Table S2 *Assembly_stats.csv*). These quality metrics indicate the successful reconstruction of microbial scaffolds, ensuring a solid foundation for the subsequent metagenomic analysis.

### 3.1. Microbial Community Composition and Clinical Relevance

**Figure 2:**
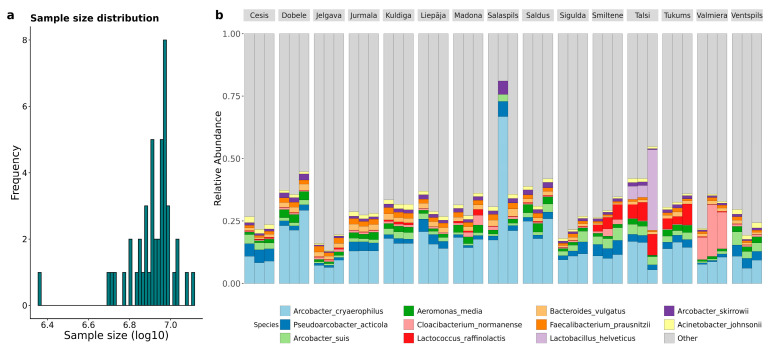
Samples before filtering outliers. (**a**) Sample size distribution of bacterial reads. The figure shows that one sample is a clear outlier, which comes from Salaspils.2. (**b**) Barplot of the relative abundance of species among cities, showing that Talsi and Salspils differ in species composition from other repeated samples.

Taxonomy profiles consisted of 6837 distinct bacterial operational taxonomic units (OTUs), accounting for 87% (328,643,695 reads) of all reads used in reconstruction (Table S3 *taxonomy_species.xlsx*). Taxonomic classification revealed 1389 genera and 5997 species, with 52.7% of quality-filtered reads identified to the species level. However, two samples required exclusion: Salaspils.2, which possessed adequate depth but unrepresentative classification rates affecting species detection, and Talsi.3, which was compromised by dairy wastewater discharge, causing an abnormal *Lactobacillus helveticus* spike (Figure 2). Following the removal of outliers and singletons, a robust dataset of 6798 OTUs and 361,540,544 sequences was retained.

**Figure 3:**
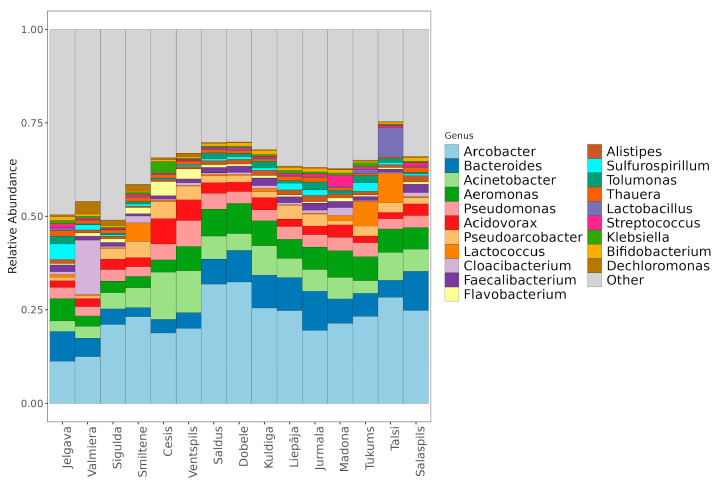
Relative taxonomic composition at the genus level per municipality.

The municipal wastewater microbiome is characterized by a stable core of anthropogenically associated genera, although some municipalities displayed distinct taxonomic profiles driven by local activities. The community was dominated by *Arcobacter* (22.25% ± 6.72%), *Bacteroides* (6.48% ± 2.57%), *Acinetobacter* (6.09% ± 2.98%), and *Aeromonas* (5.52% ± 1.82%), with *Arcobacter cryaerophilus* (15.36% ± 9.60%) identified as the most abundant species overall (Figure 3). Deviations from this core were observed in Valmiera, where *Cloacibacterium normanense* (14.74% ± 5.89%) replaced *Arcobacter* as the dominant species, and in Madona, which exhibited elevated levels of *Streptococcus* (5.78% vs. mean 0.69%) and *Cloacibacterium*. Additionally, *Lactococcus raffinolactis* showed increased abundance in Smiltene (4.42%), Tukums (5.23%), and Talsi (7.09%) compared to other cities. While the high prevalence of *Arcobacter* reflects the general anthropogenic nature of the environment, the dominance of unique taxa like *Cloacibacterium* in Valmiera highlights how specific local industrial or environmental factors shape city-specific microbial fingerprints.

Within the 78 identified genera, we observed multidrug-resistant ESKAPE pathogens [78] at the species level, as well as genera potentially containing these organisms, across all samples (Table 2). Distinct microbial patterns appeared in hospital-hosting cities (Tukums, Cesis, and Jelgava), where bacteria from the genus *Raoultella* (0.41% ± 0.89%) were detected, alongside increased *Klebsiella* abundance in Cesis (0.70%). High-virulence species such as *Acinetobacter baumannii* (0.15% ± 0.086%) and *Pseudomonas aeruginosa* (0.19% ± 0.07%) were also consistently identified across municipalities. The presence of these specific genera and species underscores the role of municipal wastewater as a critical reservoir for high-risk pathogens.

**Figure 4:**
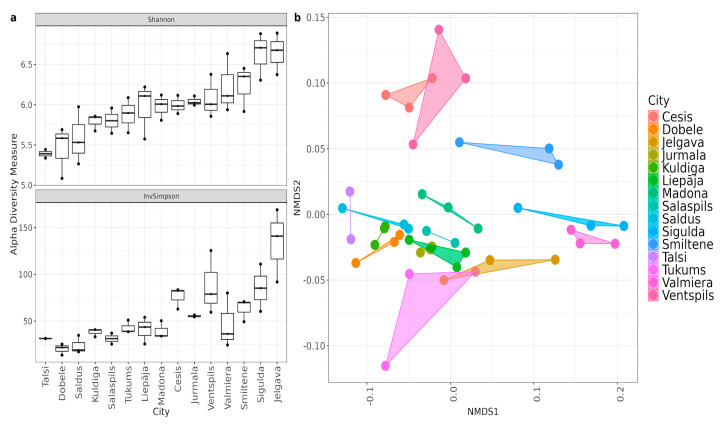
(**a**) species diversity between cities. (**b**) Species beta diversity plot. Non-metric multidimensional scaling (NMDS) ordination from Bray–Curtis distances with the resulting ordination stress value 0.099.

Geographic location was found as the primary driver of microbial community structure, creating statistically significant variations in both species richness and composition among municipalities (Figure 4). Alpha diversity metrics showed variance, with the Shannon index (Kruskal–Wallis χ^2^ = 29.15, *p* = 0.01) and Inverse Simpson index (χ^2^ = 34.69, *p* = 0.002) revealing significant differences in evenness. Post hoc testing distinguished Jelgava, Saldus, and Sigulda from other municipalities, with specific pairwise differences noted between Jelgava and Talsi (*p* = 0.028) and Dobele and Sigulda (*p* = 0.038). Beta diversity analysis further confirmed spatial clustering, as PERMANOVA indicated that municipality location explained approximately 83% of the total variation (*p* = 0.001). The stability of these clusters, confirmed by low intragroup variability (Beta dispersion F = 0.60, *p* = 0.84), demonstrates that local urban infrastructure generates persistent profiles rather than random community fluctuations.

### 3.2. Environmental Resistome Profile

**Figure 5:**
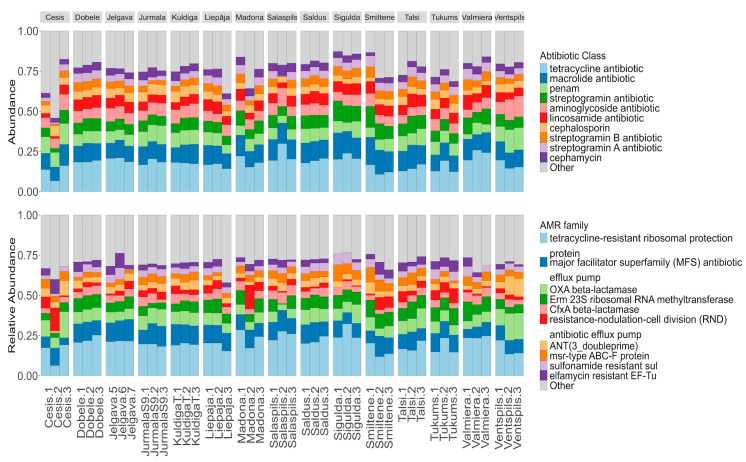
Top 10 ARGs and Resistance gene groups. Relative abundance of reads found in contigs.

The metagenomic analysis identified a diverse array of ARGs, dominated by tetracycline resistance determinants but also containing critical last-resort mechanisms. A total of 417 distinct ARGs belonging to 108 gene families and conferring resistance to 29 distinct drug classes were identified (Figure 5). The most abundant ARG in the resistome was tetracycline resistance, with the ribosomal protection gene *tet* (*Q*) being the single most abundant ARG across all samples (11.22% ± 2.79%). Beyond tetracyclines, the most frequently observed resistance determinants included macrolide resistance genes (*ErmB* 4.71% ± 1.97%, *msrE* 4.47% ± 1.99%), sulfonamide resistance genes (*Sul1* 3.58% ± 1.29%), and beta-lactamases (*CfxA6* 2.88% ± 0.88%). Functional annotation revealed that the resistance mechanisms were primarily dominated by major facilitator superfamily antibiotic efflux pumps, ribosomal protection proteins, RND efflux systems, and OXA-type beta-lactamases. We detected a low but widespread distribution of mobilized colistin resistance *MCR phosphoethanolamine transferase genes* in Jelgava, Cesis, Smiltene, and Madona, with relative abundances ranging from 0.64% to 3.16% in specific samples (Table S4 *output_drugClasses.xlsx*). 

**Figure 6:**
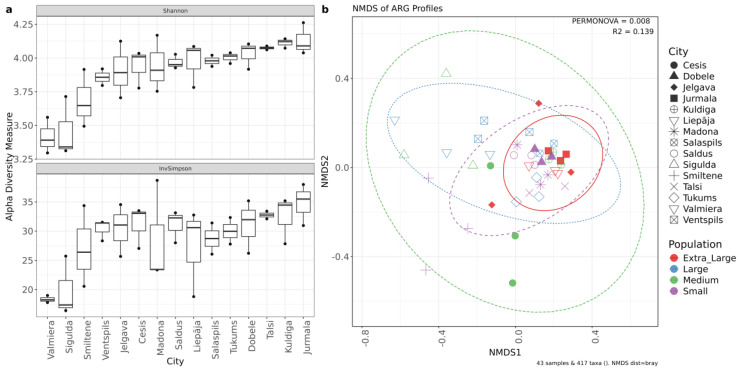
(**a**) Alpha diversity indices of ARG counts in cities by Shannon, InvSimpson, sorted from lowest to highest by Shannon values. (**b**) NMDS plot of Bray–Curtis distances between samples. Samples are colored by the size of the connected population and municipalities are represented by different shapes.

Similar to the bacterial community, municipal identity was the primary determinant of the resistome, explaining the majority of the variation in ARG composition. Alpha diversity analysis suggested that while the diversity of ARGs varied significantly by location (Shannon χ^2^ = 28.42, *p* = 0.013), the relative abundance patterns of dominant ARGs remained consistent (Inverse Simpson *p* = 0.15) (Figure 6). Post hoc Dunn’s test revealed significant differences between municipality pairs, with Jurmala and Kuldiga both showing statistically significant differences to Sigulda (*p* = 0.024 and *p* = 0.030, respectively) and Valmiera (*p* = 0.023 and *p* = 0.043, respectively). Municipality location accounted for 65.87% of the variation in ARG communities (PERMANOVA R^2^ = 0.66, *p* = 0.001), while we could not prove that population size served as a significant factor (Figure 6).

Association testing with SIAMCAT identified statistically significant (*p* adjusted < 0.05) city-specific ARG profiles:

• Cesis showed differential abundance of both efflux and aminoglycoside resistance genes (aadA6/aadA10, oqxA), Mycobacterium tuberculosis rpsL mutations, and Klebsiella pneumoniae variants (KpnE, OmpK37, acrA),
• Tukums exhibited elevated oqxB, Klebsiella pneumoniae KpnF, APH(3)-IIIa, SAT-4, FOX-5, LCR-1, ramA, and QnrD1,
• Talsi’s profile contained FOX-2 and QnrD1,
• Smiltene features tet (B) with its regulator tetR.
• Sigulda uniquely demonstrated IMP-13 metallo-β-lactamase,
• Saldus aadA15, and Salaspils OXA-140 carbapenemase.
• Kuldiga displayed the broadest spectrum including AAC(6)-Ib7, aadA4, OXA-368, APH(3)-Ia, FOX-5, GES-7, MOX-3, dfrB3, and the bifunctional AAC(6)-Ie-APH(2)-Ia.
• Jurmala contained RSA1-1 and YajC, whereas Dobele showed tet(H), Erm(35), tet(T), catB8, and floR.

### 3.3. Hospital Impact on Wastewater Resistance Genes

To assess healthcare contributions to municipal AMR, we first examined the impact of major regional hospitals on ARG diversity in WW systems. Contrary to expectations, municipalities with and without regional hospitals showed no significant differences in ARG or bacterial diversity (Wilcoxon *p* > 0.05). This result led us to investigate a more nuanced classification of healthcare institutions, as most municipalities contained smaller healthcare facilities that might contribute to AMR pollution through indirect pathways. Analysis by healthcare institution type revealed significant differences in alpha diversity of WW resistomes. While bacterial diversity was different in both evenness and richness, ARGs were only significantly different in evenness. Hospital classification (e.g., specialized facilities, Type 2-4 hospitals, Red Cross hospitals, and branch institutions) explained substantial variation in ARG community composition (PERMANOVA R^2^ = 0.364, F = 2.86, *p* < 0.001). This suggests that healthcare facility specialization and operational scope, rather than mere presence, determine AMR contributions to municipal WW.

Post hoc analysis identified distinct resistome signatures among hospital types. Type 3 hospitals (medium-complexity facilities) showed the strongest differentiation from specialized hospitals in ARG diversity (Dunn’s z = 3.14, *p* = 0.024), with additional significant differences from Type 4 hospitals (z = 2.69, *p* = 0.049) and Red Cross facilities (z = 2.51, *p* = 0.042). Similar patterns emerged for bacterial community diversity, with specialized hospitals consistently differing from branch facilities (z = −3.10, *p* = 0.027) and other hospital types.

### 3.4. The Hospital-Associated Core Resistome

To understand differences in gene profiles between hospital-associated and non-hospital municipal WW systems, we did microbiome comparison between these gene and taxa sets. Out of the 6798 bacterial taxa identified across all samples, 658 constituted the global core microbiome, with 621 taxa shared between hospital and non-hospital sites. However, 73 core taxa were unique to hospital-influenced WW, compared to only 18 unique to non-hospital municipalities, indicating enrichment of specific bacterial populations in healthcare settings. Similarly, among the 456 ARGs detected, 53 formed the core resistome, with 42 genes shared across both environments. Notably, hospital WW harbored 19 unique core ARGs versus only 6 in non-hospital sites, demonstrating a substantially enriched resistance gene repertoire in healthcare-associated effluents.

While the general core resistome (shared genes) was large, cities with hospital wastewaters contained 19 unique core ARGs, compared to only 6 unique genes in non-hospital sites. This hospital-specific ARGs consisted of high-priority, clinically relevant resistance mechanisms, including:

• Beta-lactamases: OXA-type genes (*OXA-205*, *OXA-20*) and carbapenem-hydrolyzing enzymes (*CblA-1*),
• Aminoglycoside resistance: Multiple modifying enzymes (*ANT(6)-Ia*, *ANT(2″)-Ia*, *AAC(6′)-Ib9*),
• Fluoroquinolone resistance: Plasmid-mediated determinants (*QnrS2*) and efflux pumps (*AcrF*, *acrB*),
• Macrolide and Trimethoprim resistance: *EreA2*, *mel*, *dfrA14*, and *dfrF*.

Associations analysis (SIAMCAT *p* < 0.05) further linked specific hospital types to distinct genes in comparison to the rest of the group:

• Type 2 hospitals with the following ARGs: oqxB, APH(3)-IIIa, FOX-5, Klebsiella pneumoniae KpnF, QnrD1, and ramA regulatory genes,
• Red Cross hospitals showed enrichment of tet (B) and tetR genes,
• Specialized hospitals with IMP-13, and type 3 hospitals with fluoroquinolone resistance in A. baumannii.

This hospital-unique ARG profile represents high-priority resistance mechanisms commonly associated with healthcare-associated infections and multidrug-resistant pathogens, highlighting hospitals as concentrated sources of clinically relevant AMR in municipal WW networks.

### 3.5. Industrial Factors Shape Resistance Gene Composition

Among industrial factors, the food industry emerged as the strongest differentiator of wastewater composition between samples, significantly impacting both bacterial taxonomy (R^2^ = 0.169, *p* = 0.024) and ARG profiles (R^2^ = 0.168, *p* = 0.01). Industrial wastewater discharge overall was a significant indicator of group differences, with the food industry sector contributing most substantially to this variation. Other sectors, including metal processing and meat production, showed no significant impact on the resistome profile between groups. The only metric that significantly impacted resistance gene alpha diversity of the sample was the car washing facilities.

The direct impact of food production was clearly demonstrated in the municipality of Talsi. During the sampling period, a specific industrial discharge event from a dairy production facility was captured. This event caused a massive spike in *Lactobacillus helveticus* relative abundance (making the sample an outlier for general analysis). Given the extreme deviation caused by this event, sample Talsi.3 was identified as a statistical outlier. It was excluded from the general beta-diversity and correlation analyses to prevent the skewing of global municipal trends. However, rather than discarding the data point entirely, it was retained for this specific case to investigate the direct impact of industrial discharge on the resistome. Crucially, this taxonomic shift was accompanied by a unique resistance signature: the appearance of *Propionibacterium 23S rRNA mutations conferring macrolide resistance*. This provides direct evidence that specific industrial discharges can introduce niche-specific resistance determinants into the municipal wastewater system linked to the dairy industry.

### 3.6. ARG Occurrence in Plasmids and MAGs

**Figure 7:**
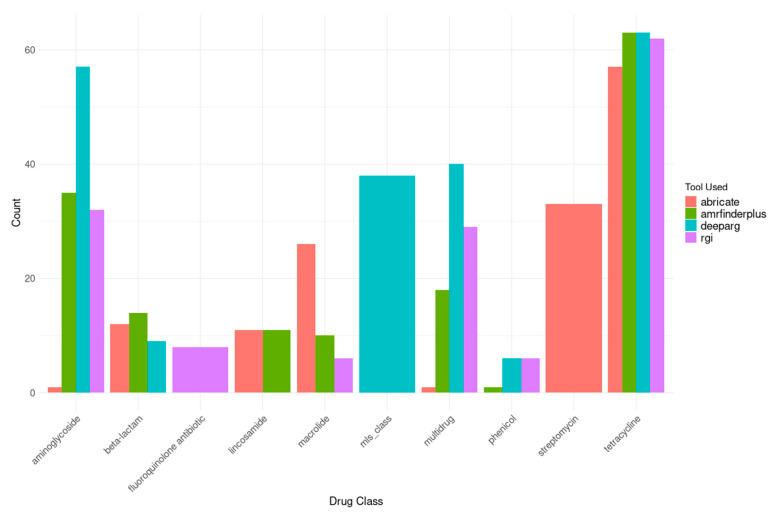
Top 10 most common Drug classes and genes found in plasmids, analyzed using multiple tools in the FuncScan pipeline with duplicates.

Analysis of the mobilome revealed a substantial reservoir of horizontal transfer elements, with 9501 plasmid sequences identified across Latvian municipal wastewater. These plasmids contained 85 unique ARGs with high sequence identity (>90%). The funcscan results combined overlapping findings from multiple tools: deeparg (236 gene locations), RGI (160 gene locations), AMRfinderplus (158 gene locations), and abricate (156 gene locations) (see Figure 7).

The plasmid-associated resistome contigs consisted of multidrug-resistant genes (88 occurrences) and clinically critical resistance classes, including aminoglycosides (125 counts) and tetracyclines (245 combined counts) (Table S5 *plasmid_genes.xlsx* and Table S6 *plasmids_funcscan.gtf*). Crucially, last-resort colistin resistance determinants *mcr-10.1* and *mcr-9.1* were identified within reconstructed plasmid sequences, indicating that these high-risk genes are present in mobile elements.

To determine the chromosomal and genomic context of these resistance markers, we reconstructed 210 dereplicated Metagenome-Assembled Genomes (MAGs). Among the reconstructed MAGs, we found that 37 of 210 dereplicated genomes (17.6%) contained ARGs. In total, the 210 reconstructed bins belonged to 151 taxa according to the GTDB database or 139 taxa based on NCBI majority vote classification (Table S7 *MAGS_taxonomy.tsv* and Table S8 *hamronization_MAGs_report.tsv*). Notably, resistance genes were concentrated within genera comprising opportunistic pathogens frequently associated with healthcare-associated infections, including *Pseudomonas*, *Acinetobacter*, *Klebsiella*, *Citrobacter*, *Aeromonas*, *Enterococcus*, *Streptococcus*, and *Clostridium*.

Identical genes were detected in both plasmids and genomes. We identified 41 ARGs present in both MAGs and plasmids, indicating bacteria and plasmids carrying identical resistance genes. Of these shared genes, 20 were annotated as multidrug resistant, followed by six beta-lactam resistance genes, five tetracycline resistance genes, six macrolide resistance genes, and three MLS-class resistance genes. The presence of clinically important ARGs shows that bacteria and mobile elements carry potential multidrug and clinically critical resistance determinants.

## 4. Discussion

This metagenomic analysis of 15 Latvian municipalities demonstrates that urban WW systems function as geographically differentiated reservoirs of AMR. Our findings reveal that resistome distinctions persist even at the local municipal scale, aligning with global evidence of geographic structuring [11,79]. However, we observed a divergence between resistome richness and evenness. While local conditions influenced the presence of rare ARGs, the relative abundance of dominant resistance determinants remained stable across municipalities. This stability suggests that urban WW systems function as selective ecosystems where “core” ARGs persist despite fluctuations in the underlying bacterial community caused by local environmental factors.

The influence of healthcare on the resistome appears driven by institutional specialization rather than the simple presence of a regional hospital. Cities hosting specialized facilities were distinguished by high-risk markers, such as *IMP-13 carbapenemases*. However, since this study relies on municipal-level wastewater sampling and limited group size, we can only hypothesize a clinical origin. We cannot definitively rule out the contribution of other unmeasured local factors to the presence of these genes. Furthermore, the general resistome profile reflected national antibiotic consumption patterns. The widespread presence of the *tet* (*Q*) gene aligns with European Medicines Agency reports identifying tetracyclines as the highest-selling antimicrobials in Latvia [80]. Additionally, the detection of *mcr phosphoethanolamine transferase genes* in cities like Jelgava and Cesis indicates the circulation of plasmid-mediated colistin resistance, highlighting the utility of wastewater surveillance in tracking the environmental dissemination of restricted antibiotics.

Food production WW emerged as the strongest industrial driver of both bacterial taxonomy and ARG profiles, with the Talsi dairy discharge event providing compelling evidence for industry-specific surveillance. The unique detection of *Propionibacterium freudenreichii* and associated macrolide resistance genes exclusively in Talsi samples, alongside elevated *Lactobacillus abundance*, demonstrates direct traceability between industrial discharges and municipal resistome alterations. These findings position food processing facilities as specific contributors to urban AMR reservoirs.

A crucial insight of this study is that ARG profiles displayed higher similarity across sites than bacterial communities, suggesting that resistance genes may be uncoupled from their taxonomic hosts. This potential for mobilization was reflected in the identification of identical resistance genes in both mobile elements and chromosomal MAGs of multidrug-resistant opportunistic pathogens, such as *Acinetobacter*, *Pseudomonas*, and *Arcobacter*, which are capable of thriving in environmental, wastewater, and clinical contexts [5]. This leads us to suggest that municipal wastewater systems may function as evolutionary hubs, providing a reservoir of mobilizable resistance strategies available to diverse bacterial populations

Despite detecting clinically relevant bacteria and resistance genes, the specific transmission pathways remain unclear. First, the origin of detected ARGs remains obscure. It is difficult to determine whether detected levels and repertoire are typical for the studied environment or directly influenced by the studied industrial factors. A primary limitation is the small sample size within factor groups and the low number of replicates per municipality. Based on the current analysis, it is difficult to draw definitive conclusions regarding specific factor influences, since new samples could drastically change the results of statistical analysis. Other longitudinal studies have shown that seasonal climatic changes can significantly alter wastewater microbiomes [23]. Longer sampling periods would allow us to differentiate if the same factors are consistent throughout the year. Finally, metagenomic approaches are strictly dependent on reference databases (e.g., CARD), which are biased toward culturable bacteria with clinical relevance. As observed with the FuncScan pipeline, different resistance gene detection tools do not produce the same results. Metagenomic assembly is still one of the bottlenecks in the identification of mobile genetic elements [81], limiting our ability to fully map resistance gene transfer between bacteria.

## 5. Conclusions

This metagenomic analysis revealed that municipal WW systems have unique microbiome signatures that shape the local ARG repertoire. We observed that microbial composition is more influenced than ARG composition by different factors, suggesting that resistance genes are linked to the broader community rather than specific taxa. While population density demonstrated only a marginal effect, we did not detect a significant correlation between ARG distribution and the mere presence of regional hospitals. These results set a baseline for the resistance and microbial background found in WW withinthis region.

Although this study provides valuable insights into region-specific ARG diversity, the underlying mechanisms that are driving these differences remain to be fully defined. The dominance of specific bacterial species and ARGs requires further investigation to understand their selective advantages. While our metagenomic approach generated comprehensive data on the ARG repertoire, complementary methods are needed to fully elucidate these complex relationships. Further research should focus on longitudinal studies across different geographical locations and temporal scales to validate the influence of the identified factors. Such extended investigations would help to establish whether these patterns are consistent across different urban environments and seasons in Latvia, ultimately contributing to our understanding of the evolution and dissemination of antibiotic resistance.

## Figures and Tables

**Table 1:** Municipalities grouped by population size.

City	Population Category	Population Range
Smiltene	Small	Up to 10,000
Madona	Small	Up to 10,000
Talsi	Small	Up to 10,000
Kuldīga	Small	Up to 10,000
Saldus	Medium	10,001–16,000
Dobele	Medium	10,001–16,000
Sigulda	Medium	10,001–16,000
Tukums	Medium	10,001–16,000
Cesis	Medium	10,001–16,000
Salaspils	Large	16,001–35,000
Valmiera	Large	16,001–35,000
Ventspils	Large	16,001–35,000
Jelgava	Extra Large	Over 35,001
Liepāja	Extra Large	Over 35,001
Jūrmala	Extra Large	Over 35,001

**Table 2:** Highlighted bacteria with clinical relevance and high virulence. Mean relative abundance and standard deviation of genera and species identified in the sample set.

Genus/Species	Mean Relative Abundance	Genus/Species	Mean Relative Abundance
*Klebsiella*	0.696% ± 0.930%	*Citrobacter*	0.587% ± 0.782%
*Klebsiella pneumoniae*	0.1547% ± 0.1785%	*Citrobacter freundii*	0.147% ± 0.140%
*Klebsiella huaxiensis*	0.0699% ± 0.0648%	*Citrobacter portucalensis*	0.108% ± 0.280%
*Acinetobacter*	6.086% ± 2.975%	*Citrobacter braakii*	0.019% ± 0.010%
*Acinetobacter baumannii*	0.150% ± 0.086%	*Aeromonas*	5.516% ± 1.818%
*Acinetobacter johnsonii*	1.213% ± 0.525%	*Aeromonas caviae*	0.299% ± 0.158%
*Pseudomonas*	3.554% ± 1.313%	*Aeromonas dhakensis*	0.012% ± 0.005%
*Pseudomonas aeruginosa*	0.194% ± 0.073%	*Aeromonas veronii*	0.821% ± 0.432%
*Pseudomonas alcaligenes*	0.275% ± 0.157%	*Enterobacter*	0.285% ± 0.276%
*Escherichia coli*	0.268% ± 0.101%	*Enterobacter cloacae*	0.100% ± 0.225%
*Staphylococcus aureus*	0.006% ± 0.002%	*Enterococcus faecium*	0.052% ± 0.024%

## Data Availability

The Whole Metagenome Shotgun Sequencing Data is available in the ENA repository and can be accessed via the following DOI: https://identifiers.org/ena.embl/PRJEB79273c (accessed on 5 January 2026). The Metagenome Assembled Genome (MAG) Assemblies are also stored in the ENA repository and can be accessed using the DOI: https://identifiers.org/ena.embl/PRJEB80484 (accessed on 5 January 2026). Supplementary Materials are available under Zenodo DOI accession 10.5281/zenodo.17912239. A full record of all statistical analysis is included as Additional file (Publication_ARG.pdf, Publication_tax.pdf and Publication_mobilome.pdf), and were created using the *knitr* package in R. R analysis scripts are available in GitHub (https://github.com/EdgarsLiepa/wastewater_LV (accessed on 5 January 2026)). Taxonomic Classification Data is available in the Supplementary Information, specifically in the file waste_water.biome. AMR Classification Data is available in the Supplementary Information, specifically in the file AMR_genes_RGI_scaffolds_filtered.csv, hamronization_MAGs_report.tsv and hamronization_combined_report_fixed_names.tsv. Metadata is available in the Supplementary Information, specifically in the file sampleMetadata.csv.

## Supplementary Materials

The following supporting information can be downloaded at: https://www.mdpi.com/article/10.3390/microorganisms14010145/s1, Table S1: *sequencing_reads_par_sample.tsv*; Table S2: *Assembly_stats.csv*; Table S3: *taxonomy_species.xlsx*; Table S4: *output_drugClasses.xlsx*; Table S5: *plasmid_genes.xlsx;* Table S6: *plasmids_funcascan.gtf*; Table S7: *MAGS_taxonomy.tsv*; Table S8: *hamronization_MAGs_report.tsv.*

## Abbreviations

The following abbreviations are used in this manuscript:

ARG	Antimicrobial resistance genes
AMR	Antimicrobial resistance
CARD	Comprehensive Antibiotic Resistance Database
CSS	Cumulative Sum Scaling
EMBL-EBI	European Bioinformatics Institute
FIII	Food Industry Impact Index
HGT	Horizontal gene transfer
NMDS	Non-metric Multidimensional Scaling
NGS	Next generation sequencing
MAGs	Metagenome Assembled Genomes
OTUs	Operational taxonomic units
WBE	WW-based epidemiology
WHO	World Health Organization
WW	Wastewater
WWTP	WW treatment plant
